# Screening and hit evaluation of a microbial metabolite library against the pathogenic *Plasmodium falciparum* and *Toxoplasma gondii* parasites

**DOI:** 10.1016/j.ijpddr.2025.100606

**Published:** 2025-08-05

**Authors:** Maria R. Gancheva, Emma Y. Mao, Ornella Romeo, Daniel Vuong, Ryan O'Handley, Stephen W. Page, Ernest Lacey, Danny W. Wilson

**Affiliations:** a Research Centre for Infectious Diseases, School of Biological Sciences, The University of Adelaide, Adelaide, 5005, South Australia, Australia; b ARC Training Centre for Environmental and Agricultural Solutions to Antimicrobial Resistance (CEAStAR), St Lucia, 4072, Queensland, Australia; c School of Animal and Veterinary Sciences, The University of Adelaide, Roseworthy, 5371, South Australia, Australia; d Microbial Screening Technologies Pty Ltd, Smithfield, 2164, New South Wales, Australia; e Neoculi Pty Ltd, Newtown, 2042, New South Wales, Australia; f Burnet Institute, Melbourne, 3004, Victoria, Australia

**Keywords:** Drug discovery, Natural products, Malaria, Toxoplasmosis

## Abstract

Frontline drug treatments for malaria are at risk of failing due to emerging resistance, meanwhile drugs used to treat toxoplasmosis have suboptimal efficacy and safety. As demonstrated by the success of clinically used antiparasitic drugs, the diverse structural complexity and biological activity of natural products holds great potential for drug discovery and development, to address the need for new compounds with novel mechanisms. Here we screened the BioAustralis Discovery Plates Series I library, a collection of 812 microbial natural product compounds including rare microbial metabolites, against *Plasmodium falciparum* erythrocytic stage and *Toxoplasma gondii* tachyzoite parasites. We identified 219 compounds that inhibited *P. falciparum* growth by at least 80 % at a concentration of 2 μg/mL (1–10 μM for >90 % of compounds), whilst 149 compounds demonstrated equivalent activity against *T. gondii*. The active compounds were assigned based on chemical structure to more than 50 compound classes. After triaging active compounds for those with low mammalian cytotoxicity, we defined the *in vitro* half maximal inhibitory concentration (IC_50_) of a selection of compounds against the parasites, identifying four compound groups with activity in the low nanomolar range. The macrocyclic lactone pladienolide B and cryptopleurine were found to be very potent against the parasites but also mammalian cells, warranting further structure-activity relationship investigation. Two groups, the monocyclic thiazole peptides, including micrococcin P1 and the thiocillins, and the pleuromutilins, exhibited both low antiparasitic IC_50_ and low cytotoxicity, highlighting their potential for further analysis. This study defines the activity of the BioAustralis Discovery Plates Series I against two apicomplexan parasites of significant global importance, providing potential new tools to study parasite biology and possible starting points for novel antiparasitic development.

## Graphical abstract

**Figure:**
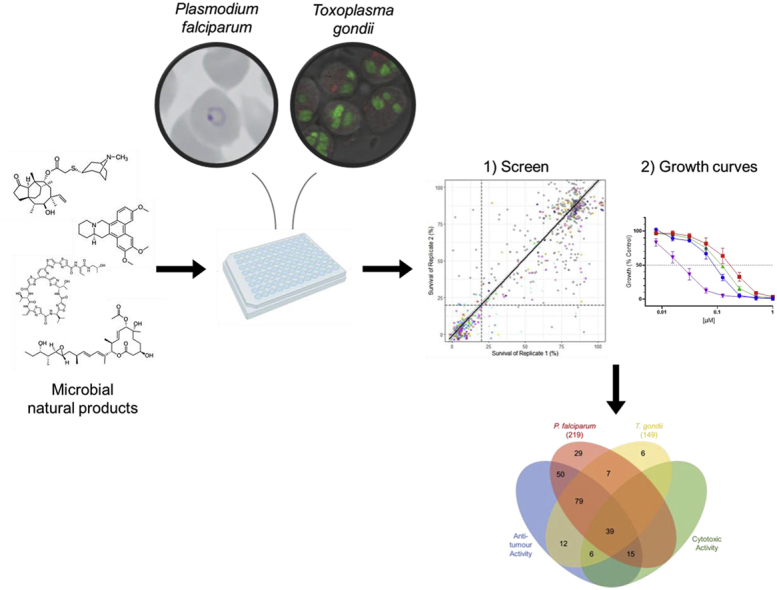

## 1 Introduction

The apicomplexan phylum of single-celled, obligate intracellular parasites, comprises more than 6000 known species, many of which account for significant global human and animal disease burden. The *Plasmodium* and *Toxoplasma* genera represent the most medically-relevant and best-studied members of this phylum, responsible for causing malaria and toxoplasmosis, respectively.

Malaria is a mosquito-borne infectious disease, whereby female *Anopheles* mosquitoes are responsible for transmitting the disease-causing parasites. Five species within the *Plasmodium* genus cause malaria in humans of which *P. falciparum* is the most virulent and the predominant species in Sub-Saharan Africa (World Malaria Report, 2023). *Plasmodium* spp. cause more than 200 million cases of malaria and result in over 600,000 deaths each year, most of which are in children under the age of 5 years (World Malaria Report, 2023). Additionally, malaria, which impacts 10 million pregnancies every year (World Malaria Report, 2023), may cause severe disease in the mother and result in poor developmental outcomes for the baby (Rogerson et al., 2018). Artemisinin-based combination therapies, the frontline treatment for malaria, and the increased distribution of insecticide-treated bed nets have significantly reduced malaria cases and deaths over the last 25 years (World Malaria Report, 2023). Unfortunately, resistance to antimalarial drugs has developed and spread throughout Southeast Asia and has emerged in Africa and South America (Amaratunga et al., 2016; Dondorp et al., 2009).

*Toxoplasma gondii*, a ubiquitous food- and water-borne zoonotic parasite, can infect a wide range of hosts. It is one of the most successful parasites, estimated to chronically infect more than a third of the human population at any given time, with local prevalences within some areas of Latin America reaching 90 % (Pappas et al., 2009). Each year, *T. gondii* is estimated to impact 1.5 out of every 1000 live births (190,000 cases) globally, leading to foetal loss, birth defects or developmental problems (Li et al., 2014; Torgerson and Mastroiacovo, 2013). It is further associated with ocular disease, cerebral infection in immunocompromised individuals, and chronic physical and mental health problems (Basavaraju, 2016; Ferreira et al., 2022). The current treatment options for acute toxoplasmosis, utilising old drugs that are prescribed off-label, aim to minimise clinical disease and long-term sequelae. The antimalarial drug pyrimethamine is a standard component, typically combined with a sulfonamide antibiotic (*e.g.* sulfadiazine, sulfadoxine), together inhibiting the folate pathway and DNA synthesis, and therefore supplemented with folinic acid (Dunay et al., 2018; Wei et al., 2015). An alternative treatment regimen using the antibiotic trimethoprim and sulfamethoxazole also targets this pathway (Dunay et al., 2018). Other drugs that may be included in combination therapy, in the absence of better alternatives, include the antimalarial atovaquone and antibiotics, such as clindamycin and azithromycin (Dunay et al., 2018; Wei et al., 2015). The antibiotic spiramycin is used to prevent foetal transmission, but is ineffective for established foetal infections, and with pyrimethamine's teratogenicity rendering it unsafe in the first trimester, treatment options for active foetal infections in the first trimester are limited (Milewska-Bobula et al., 2015; Wei et al., 2015). Overall, these treatments are considered to have serious safety concerns, limited efficacy, emerging resistance and are ineffective against chronic infections.

Given the impact of *P. falciparum* and *T. gondii* parasites on human health and the issues associated with current control and treatment strategies, there is an urgent need to develop drugs of novel chemotypes that can be used for treatment and prophylaxis to improve disease outcomes threatened by emerging global resistance. More than 50 % of clinically available drugs are directly or indirectly derived from natural products from plants, animals and microbes, including most antimalarial drugs (Newman and Cragg, 2020; Siqueira-Neto et al., 2023; Tu, 2016). Since the discovery of penicillin in the 1930s (Fleming, 1929), we have recognised that within the microbiome, microbes act antagonistically to impair development, inhibit growth and kill competitors. Conversely, these same microbes can act co-operatively, constructively, combine synergistically and commensally. These and many more actions are controlled by secondary metabolites. Natural product metabolites produced by microbes represent an incredibly diverse array of structural complexity and biological activity, and have historically played a key role in drug discovery. Compounds derived from microbial metabolites, such as penicillin, azithromycin and clindamycin, remain some of the most important antimicrobial drugs ("Web Annex A. World Health Organization Model List of Essential Medicines – 23rd List, 2023.," 2023). Natural products are important sources for novel chemical scaffolds (Atanasov et al., 2021). With more than 500,000 natural products reported in the literature and less than 10 % of the world's biodiversity explored, there is a huge untapped potential for discovery (Cragg and Newman, 2005, 2013).

The unique biology of the apicomplexa forges two discrete molecular architectures superimposed within the parasites and presents a formidable chemotherapeutic challenge. Broadly, pathways to kill include: 1) targeting eukaryotic cell components, which impacts parasite replication within one 48 h cycle of development (fast-killing), and 2) targeting the apicoplast organelle of bacterial origin, resulting in replication defects becoming evident after two replication cycles (96 h) post treatment (slow-killing or delayed-death).

Here, we carry out a screen of a unique library of microbial metabolites with diverse biological activity, against blood-stage *P. falciparum* and replicative *T. gondii* tachyzoite parasites. The BioAustralis Discovery Plates Series I library is a collection of 812 known and rare microbial metabolites sourced mainly from Australian microorganisms, with some semi-synthetic and rare plant metabolites. This library contains compounds with established modes of action, some that can be used to confirm antiprotozoal activity and may present the opportunity to be repurposed. Importantly, this library also contains many metabolites with yet unexplored biological activity. Since clinically used antimalarial drugs typically have an *in vitro* potency of less than 100 nM (Wilson et al., 2013) and anti-toxoplasma drugs range between 200 and 500 nM (van der Ven et al., 1996), our aim was to identify antimalarial and anti-toxoplasma activity of compounds with an IC_50_ of less than 200 nM. Using our strategy, we identified a high hit-rate in both the search for new *P. falciparum* and *T. gondii* actives with over 200 microbial metabolites with antiparasitic activity against *P. falciparum* within one growth cycle and more than 140 with similar speed against *T. gondii*. We further explored the potency of 20 microbial metabolites and additional analogues that have little or no previously reported *in vitro* activity against malaria and *Toxoplasma* parasites.

## 2 Materials and methods

### 2.1 *In vitro* parasite and cell culture

*P. falciparum* 3D7 parasites were cultured in human O+ red blood cells (Lifeblood), in RPMI-HEPES (Gibco, 23400021) culture medium (pH 7.4, 367 μM hypoxanthine (Sigma-Aldrich), 25 μg/mL gentamicin (Gibco), 25 mM NaHCO_3_ (Sigma), 2 mM L-glutamine (Gibco), 0.5 % (v/v) Albumax II (GibcoBRL)), and maintained at 37 °C in an atmosphere of 1 % O_2_, 4 % CO_2_ and 95 % N_2_ according to established protocols (Trager and Jensen, 1976). Parasites were synchronised with 5 % (w/v) sorbitol (Sigma-Aldrich) treatment to select for ring stages.

The *T. gondii* RH strain (type I) expressing enhanced yellow fluorescent protein under a tubulin promoter (RH-Tub-eYFP) (Gubbels et al., 2003) was maintained in human foreskin fibroblast (HFF) cells, in DMEM culture medium (Gibco, 12430054), supplemented with 10 % (v/v) FBS (Bovogen), 1 % (v/v) penicillin/streptomycin (Gibco), 1 mM sodium pyruvate (Gibco), and maintained in a humidified incubator with 5 % CO_2_ at 37 °C.

### 2.2 Compounds and microbial metabolite library

The BioAustralis Discovery Plates Series I library containing 812 purified microbial metabolites was provided by BioAustralis (Sydney, Australia). A full list of microbial metabolites can be found in the supplementary information (Table S1). These metabolites were provided at 1 mg/mL in DMSO in 1 μL/well in a 96-well plate, and stored at −80 °C. Compounds were screened against the parasites in biological duplicates at 2 μg/mL. Compounds used in subsequent growth assays were provided by BioAustralis, resuspended to 10 mM stocks in DMSO, and stored at −20 °C. For dose-response curves and IC_50_ determination, 8-point 1:2 serial dilutions were performed, in two technical duplicates and a minimum of 3 biological replicates. Control compounds included DMSO, 2.5 μM pyrimethamine (fast-killing drug) and 6 nM clindamycin (slow-killing drug).

### 2.3 *P. falciparum* growth inhibition assay

*P. falciparum* growth assay protocols for measuring drug inhibition across 1-cycle (72 h treatment) and 2-cycles (120 h treatment) of intraerythrocytic growth and replication have been described previously (Wilson et al., 2015). Early ring-stage parasites were prepared in 1 % haematocrit at 1 % (1-cycle) or 0.1 % (2-cycle) parasitaemia and added to round-bottom 96-well plates with 90 μL/well. Compounds were added to a total volume of 100 μL/well. Parasite growth was measured at late trophozoite/schizont stages for both 1-cycle (72 h post treatment) and 2-cycle (120 h post treatment) assays using SYBR DNA staining. To stain for parasite DNA, supernatants were removed and well contents were resuspended in 100 μL PBS. An equal volume of 0.02 % (v/v) SYBR Safe Stain (Invitrogen) in SYBR lysis buffer (pH 7.5, 20 mM TRIS, 5 mM EDTA, 0.008 % (w/v) Saponin, 0.08 % (v/v) Triton X-100) (Dery et al., 2015) was added to the wells and mixed. After incubating for 30–60 min, plates were read using the fluorescent module (excitation, 485 nm; emission, 520 nm) of a BMG Labtech PHERAstar FS microplate reader.

### 2.4 *T. gondii* growth inhibition assay

Freshly lysed *T. gondii* tachyzoites were plated on confluent HFF monolayers in flat-bottom 96-well plates at 2 × 10^3^ parasites/well in 90 μL of FluoroBrite DMEM (Gibco, A1896701) culture medium (10 % (v/v) FBS, 1 % (v/v) penicillin/streptomycin). Compounds were added to a total volume of 100 μL/well. At completion of the 72 h incubation period, parasite growth was measured using the eYFP signal, with fluorescence (excitation, 485 nm; emission, 520 nm) read-outs obtained using a BMG Labtech PHERAstar FS microplate reader (Burns et al., 2022).

### 2.5 Cytotoxicity assays

Compound toxicity against mammalian cells was determined using cultures of human neonatal foreskin fibroblast (NFF) and NS-1 murine myeloma cells. The compounds were initially screened for evidence of cytotoxicity at 5 μg/mL against NFF and NS-1 cells, and determined to be active or inactive using a resazurin dye assay (Booth et al., 2020). Briefly, NFF and NS-1 cells were transferred to 96-well plates using 190 μL culture media containing 50,000 cells/mL in DMEM (10 % (v/v) FBS, 1 % (v/v) penicillin/streptomycin) together with 10 μL resazurin (250 μg/mL) and incubated in a humidified incubator with 5 % CO_2_ at 37 °C. The plates were incubated for 96 h during which time the positive control wells change from a blue to pink colour. The absorbance of each well was measured using Thermo Scientific Multiskan Skyhigh Microplate Spectrophotometer at 605 nm. For dose-response curves and IC_50_ determination, cultures of HFF cells were used. Cells were seeded at 1 × 10^3^ cells/well in flat-bottom 96-well plates and incubated for 24 h. Compounds were added to a final volume of 100 μL and incubated for 72 h. CellTitre-Glo (Promega) was used to quantitate ATP levels as a measure of cell growth, as per the manufacturer's instructions. Briefly, 30 μL of the reagent was added to each well and mixed to induce cell lysis and release ATP. Plates were incubated for 10 min to allow the luminescent signal to stabilise, and luminescence was measured using a BMG LabTech PHERAstar FS microplate reader's luminescent module.

### 2.6 Data analysis

For antiparasitic growth assays, technical duplicates were averaged, the background (non-infected cells) was subtracted from all samples, and compound-treated parasites were normalised against untreated parasites for each 96-well plate. For cytotoxicity assays, compound-treated cell growth was normalised to non-inhibitory controls (medium only). In microbial metabolite library screens, compounds were considered a ‘hit’ or active if they displayed >80 % growth inhibition relative to the control in biological duplicates. Data visualisation of inhibitory activity of compounds was done with the ggplot2 (Wickham, 2016) and UpSetR (Conway et al., 2017; Lex et al., 2014) packages in R Studio. Dose-response curves and IC_50_ values were determined using GraphPad Prism (GraphPad Software) according to the recommended protocol for non-linear regression of a log-(inhibitor)-versus-response curve, and expressed as mean ± S.D.

## 3 Results

### 3.1 Activity of the BioAustralis Discovery Plates Series I library of microbial metabolites against *P. falciparum*

**Fig. 1:**
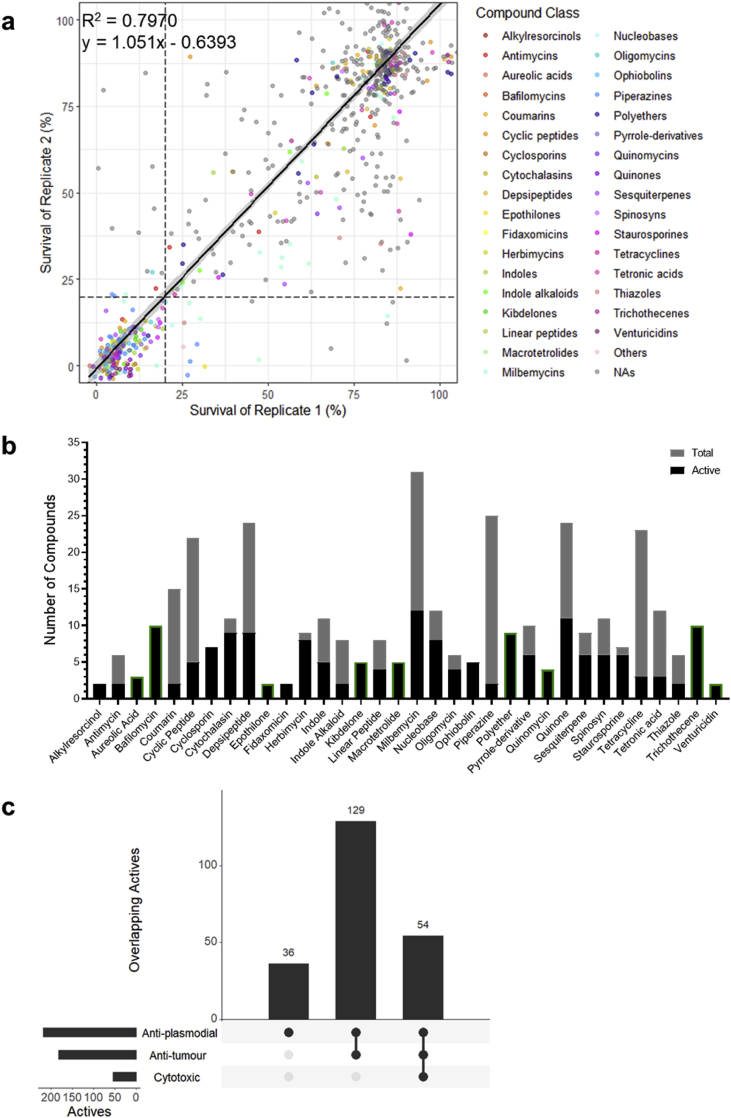
The antimalarial activity of the BioAustralis Discovery Plates Series I library of microbial metabolites. **(a)** Correlation between percentage survival of *P. falciparum* between biological duplicate screens of the microbial metabolite library. Each data point represents a single compound and dashed lines indicate 20 % survival of each replicate, used as the threshold for activity (*i.e.* active ≤20 % survival, inactive >20 % survival). Compound classes are assigned a colour if at least 2 compounds from the structural class demonstrate activity based on the average survival. Single active compounds from a class have been grouped as ‘others’, whilst non-active compounds (NAs) appear in grey. **(b)** Compound classes with at least 2 active compounds against *P. falciparum*, showing the proportion of active compounds in their classes and the total number of compounds in the respective classes within the library. Compound classes outlined in green showed the same activity in *T. gondii*. **(c)** UpSet plot depicting the overlap between antimalarial activity of microbial metabolites with anti-tumour (NS-1 cell line) and cytotoxic (fibroblast cells) activity. The filled circles on the x-axis specify the overlapping activities and the height of the bars indicate the number of overlapping active compounds. The horizontal bars represent the total number of active compounds in each screen.

We screened the 812 microbial metabolites of the BioAustralis Discovery Plates Series I library against the drug-sensitive 3D7 *P. falciparum* line using established growth conditions and assays. Compounds were screened at 2 μg/mL for quick-killing activity over one replication cycle (72 h timepoint). Replicate testing had an R^2^ value of 0.7970 and a linear curve fitted between replicate data with a slope of 1.051 (Fig. 1A). From these screens, we identified 219 compounds (27 %) that met our threshold for further exploration, with reproducible growth inhibition of *P. falciparum* of ≥80 % relative to the negative control (Fig. 1A). There was a low 2.3 % of compounds (19 compounds) that produced inconsistent growth inhibition between replicates and were cut off by the ≥80 % threshold; these were not pursued beyond the initial screen (Table S2).

The 219 active compounds belonged to 71 classes based on chemical structure (Fig. 1A). Of these 71 chemical classes, 34 classes were represented by 2 or more active compounds (Fig. 1B). These included 43 compounds across 9 subclasses of macrolides; broad-spectrum inhibitors of bacterial ribosome protein synthesis. There were 13 classes where each of the representative compounds in this library were active; these included macrolide-containing classes (bafilomycins, epothilones, fidaxomicins and venturicidins), cyclosporin and quinomycin peptides, as well as alkylresorcinols, aureolic acids, kibdelones, macrotetrolides, ophiobolins, polyethers, and trichothecenes (Fig. 1B). Amongst the active compounds were known inhibitors of *P. falciparum*, including the histone deacetylase inhibitor trichostatin A and the protein translation inhibitor cycloheximide, providing validation of our screens (Wilson et al., 2013).

To assess if any of the compounds exhibited delayed-death activity against *P. falciparum*, whereby they inhibit apicoplast biogenesis in the parasites that then leads to progeny without this organelle and cell death in the second-cycle post drug treatment, the screen included a 120 h timepoint that covered two complete replication cycles. Using known delayed-death inhibitors within the library as controls (clindamycin, azithromycin, doxycycline, spiramycin) (Dahl and Rosenthal, 2007; Pradines et al., 2001), we defined compounds that induced a delayed-death phenotype as those that: 1) exhibited ≥40 % growth at 72 h, but 2) ≤20 % growth at 120 h, with 3) a difference of at least 5-fold increase in potency from 1 to 2 cycles of intraerythrocytic replication (from 72 to 120 h) (Table S3). This resulted in the identification of 10 potential delayed-death inhibitors (Table 1).

**Table 1:** Potential delayed-death inhibitors of *P. falciparum* in the microbial metabolite library.

Compound	Structural Class	Reported Antimalarial Activity
Leucomycin A1	Macrolide (16-membered), leucomycin	Related to the clinically used and known delayed-death inhibitor spiramycin (Pradines et al., 2001). Clinically used josamycin has been predicted to target malaria parasites (Ramakrishnan et al., 2017). Clinically used tylosin and tilmicosin did not meet criterion 3 in our data. Tylosin has been reported to induce a delayed-death phenotype in malaria parasites (Arsic et al., 2018; Tsutsui et al., 2013).
Leucomycin A3 (Josamycin)		
Leucomycin A4		
Leucomycin A5		
Azamulin	Diterpene, pleuromutilin	Clinically used analogue retapamulin was predicted to target malaria parasites (Ramakrishnan et al., 2017), and induced apicoplast loss in *T. gondii* (Dos Santos et al., 2023) supporting the delayed-death activity we observed.
Tiamulin		
Dalfopristin Mesylate	Streptogramin	Derived from pristinamycin, which inhibits protein synthesis in the bacterial ribosome (Aumercier et al., 1985). Reported to act as a delayed-death inhibitor in *P. falciparum* (Barthel et al., 2008).

Novobiocin	Aminocoumarin	Targets *P. falciparum* DNA gyrase activity (Pakosz et al., 2021), hsp90 proteins (Stofberg et al., 2021) and DNA helicase activity (Ahmad et al., 2015).

Helvolic Acid	Triterpenoid	Structurally related to fusidic acid, which targets translation elongation factor proteins in malaria parasites within one life-cycle (Johnson et al., 2013).

Florfenicol	Amphenicol	Chloramphenicol has been reported to inhibit malaria parasite growth over two life-cycles (Yeo and Rieckmann, 1994).

The selectivity of the antimalarial compounds was also examined and used to determine potential compounds for further assessment. Cytotoxic and anti-tumour activity of the library compounds were based on screens against mammalian cells, foreskin fibroblasts and mouse myeloma NS-1 cell line, respectively. Comparing activity with mammalian cells is important in assessing whether a compound demonstrates evidence of potential selective toxicity to the parasites and not impacting the function of healthy mammalian host cells. Additionally, comparing activity of the active compounds with their activity in healthy cells (*e.g.* fibroblasts) and tumorigenic cells (*e.g.* myeloma cells) can provide insight into the mechanism of action. Shared antiparasitic, cytotoxic and anti-tumour activity indicates potentially conserved mechanisms of drug action across eukaryotic cells. Meanwhile, the presence of anti-tumour activity, but no cytotoxicity, may suggest shared properties in cancer cells and *Plasmodium*, both of which can modulate cellular pathways in favour of survival and rapid proliferation (Chakraborty et al., 2017).

Of the 219 antimalarial actives, 36 were selective for *P. falciparum* (Fig. 1C). These included clinically used drugs, for example; selamectin belonging to the milbemycin family of drugs that are used in veterinary medicine against parasites, fidaxomicin used against *Clostridioides difficile* colitis, and widely used broad-spectrum tetracycline antibiotics such as minocycline. There were 183 compounds that exhibited anti-tumour activity (Fig. 1C). Amongst these compounds were the nucleic acid bis-intercalators of the quinomycin family that target eukaryotic cellular replication and transcription (Kong et al., 2005; Lee and Inselburg, 1993), and the sesquiterpenoid mycotoxin trichothecenes that target translation (Ueno, 1977). A significant number of the actin polymerisation blocking cytochalasins, which have a greater impact on highly dynamic cells, were also present. Furthermore, 54 compounds with anti-tumour activity also displayed cytotoxic activity (Fig. 1C). This included several members of the quinone compound class, which have broad biological activities. Though not present in this library, this class includes the antimalarial drug atovaquone, which is known to be highly selective for the parasite electron transport system (McFadden et al., 2000; Srivastava et al., 1999). Whilst our screens indicate cytotoxic activity displayed by 25 % of the antimalarial actives at a fixed screening concentration of 5 μg/mL, some of these compounds may exhibit greater selectivity for parasites upon further examination.

### 3.2 Evaluating the potency of actives against *P. falciparum*

We next assessed the antimalarial activity for a selection of compounds active against malaria parasites across an 8-point dilution series and plotted dose-response curves to determine their IC_50_. To understand the structure-activity relationship, we also included additional structural analogues available in our in-house library, but not present in the Discovery Plates Series I library, for several compound classes where there was at least one active analogue identified in the screen (Table 2). The activities of these compounds, which have little or no prior study against malaria parasites, are summarised below.

**Table 2:**
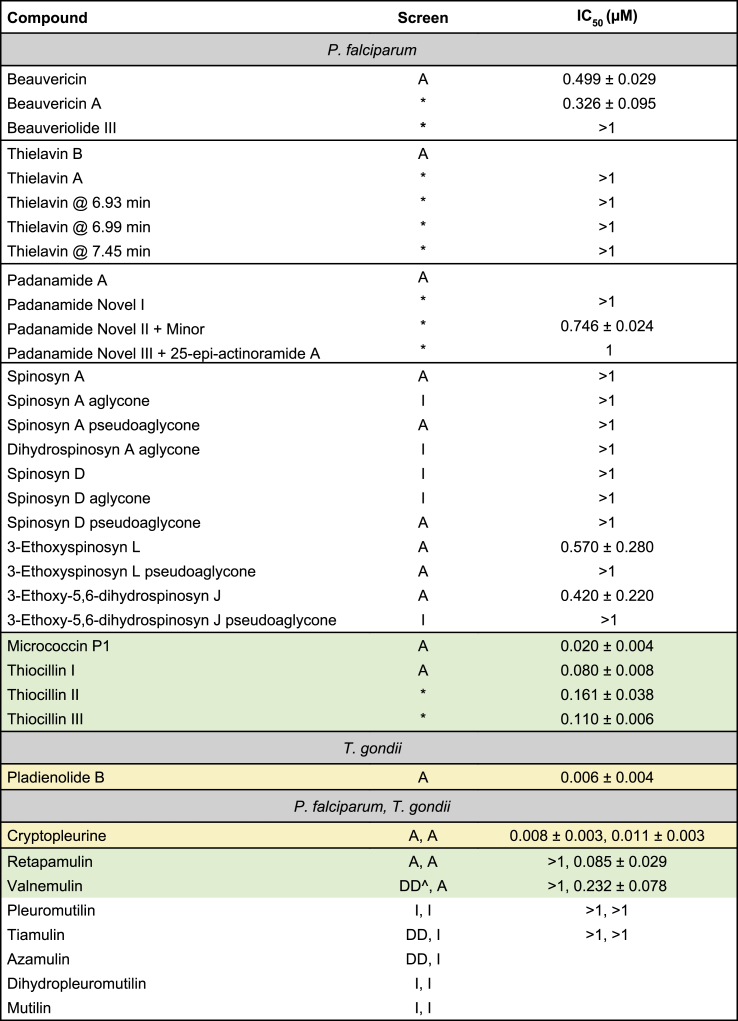
Half-maximal inhibitory concentrations (IC_50_) of a selection of ‘hit’ compounds from the screens against *P. falciparum* and *T. gondii*.

Micromolar IC_50_ values are reported as mean ± S.D., n ≥ 3. Compounds with no appreciable activity have IC_50_ > 1 μM “A”, “I” and “DD” indicate compounds that were active, inactive or exhibited delayed-death activity in the screens, respectively, while ∗ indicates analogues that were not present in the library but subsequently analysed. A blank indicates a compound that was not followed up on due to unavailability where analogues were available. ˆ indicates criteria for delayed-death activity partially met. Compounds highlighted in; yellow exhibited cytotoxicity resulting in low selectivity indices, and green exhibited low or no cytotoxicity resulting in high selectivity indices.

The cyclic depsipeptide beauvericin isolated from several fungi (*e.g. Beauveria*, *Fusarium*), which targets eukaryotic acyl-CoA:cholesterol acyltransferase responsible for converting cholesterol into cholesteryl esters, and its methyl analogue, displayed an IC_50_ of 500 and 330 nM, respectively (Table 2, Fig. S1A). The related smaller cyclic depsipeptide, beauveriolide III, was less potent with an IC_50_ greater than 1 μM (Table 2, Fig. S1A). Though these compounds were not very potent, and we did not investigate them further, they may still be relevant for alternative therapeutic strategies and as a template for further structure-activity relationship studies.

Four available analogues of the depside thielavin B were examined. All analogues exhibited an IC_50_ greater than 1 μM (Table 2, Fig. S1B). Due to the BioAustralis Discovery Plates Series I format, our initial screen was completed at 2 μg/mL. Since thielavin B has a molecular weight of 567 g/mol, it was screened at a concentration of approximately 3.5 μM where it was found to be active based on our criteria of ≥80 % growth inhibition. Therefore, it is not surprising that some compounds we identified as active at the initial 2 μg/mL did not subsequently meet our next selection criteria of an IC_50_ less than 1 μM.

The linear peptide recently isolated from marine *Streptomyces* strains, padanamide A (actinoramide A) (Nam et al., 2011; Williams et al., 2011), with a molecular weight of 662 g/mol, was active against *P. falciparum* in our screen. This compound was reported to have an IC_50_ of approximately 200 nM in *P. falciparum* in another study, however the mechanism of action has not been elucidated (Cheng et al., 2015). We further tested three novel analogues for potency, with IC_50_ values of 750 nM, 1 μM and >1 μM observed (Table 2, Fig. S1C). Interestingly, one of the analogues was a combination of padanamide A and a novel padanamide, giving it twice the molecular weight and an IC_50_ of approximately 1 μM. This suggests the parent compound imparts important activity but also highlights the potential of larger compounds and the need to further investigate this class.

This library contained 11 macrolides of the spinosyn family, compounds that are associated with disruption of nicotinic acetylcholine receptors in the nervous system and are used as insecticides (Kirst, 2010). Six of these were active against *P. falciparum* and displayed anti-tumour activity. Upon further analysis, only two of these compounds, 3-ethoxyspinosyn L and 3-ethoxy-5,6-dihydrospinosyn J, had an IC_50_ less than 1 μM (IC_50_ = 570 nM and 420 nM, respectively) (Table 2, Fig. S1D). These compounds both contain a forosamine moiety, which is essential for potent activity, and are second generation spinosyns with improved activity over the first generation spinosyns A and D (Crouse et al., 2007).

Another macrolide family, the monocyclic thiazole peptides of ribosomal origin, consisting of micrococcin P1, and three analogues, thiocillins I, II and III, exhibited IC_50_ values less than 200 nM with 72 h treatments against *P. falciparum* (Table 2, Fig. S1E). Furthermore, these monocyclic thiazole peptides did not exhibit cytotoxic activity at concentrations well above 1 μM. Micrococcin P1 was most potent with an IC_50_ of 20 nM against *P. falciparum*; it has previously been reported to have an IC_50_ of 35 nM within 48 h, though potentially targeting protein synthesis in the apicoplast (Rogers et al., 1998). Whilst apicoplast-targeting drugs typically exhibit their effects within longer treatment times, there have been reports of drugs exhibiting apicoplast-specific effects but resulting in immediate death (Uddin et al., 2018).

### 3.3 Activity of the BioAustralis Discovery Plates Series I library of microbial metabolites against *T. gondii*

**Fig. 2:**
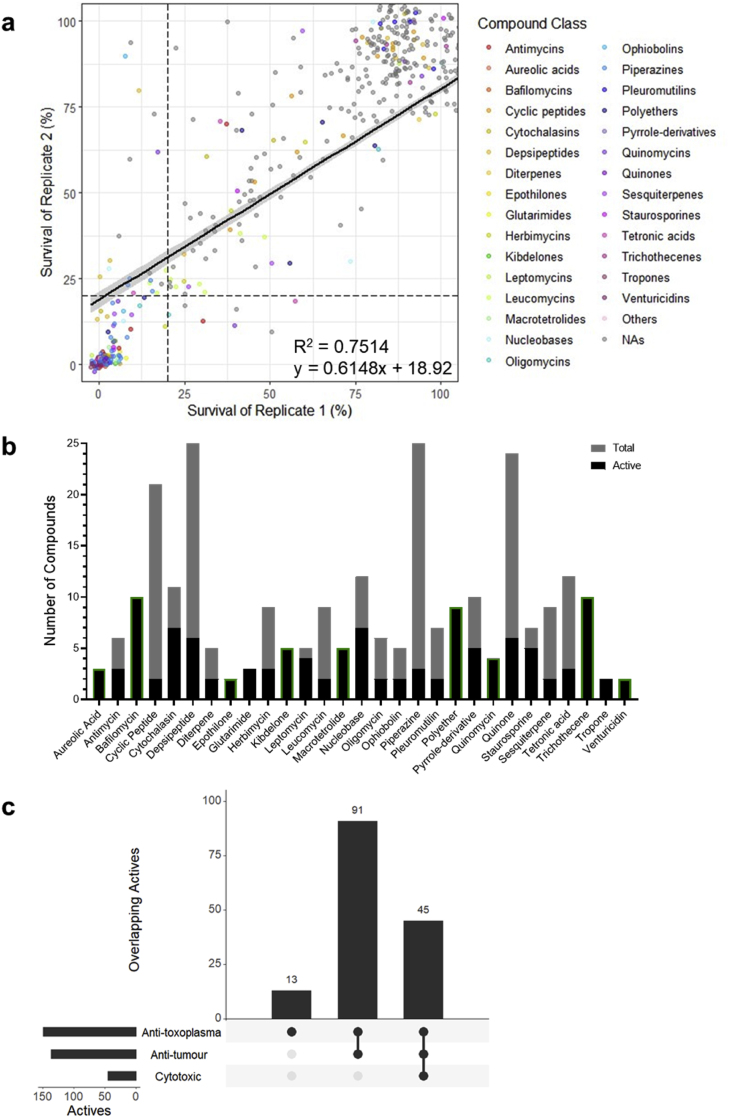
The anti-toxoplasma activity of the BioAustralis Discovery Plates Series I library of microbial metabolites. **(a)** Correlation between percentage survival of *T. gondii* between biological duplicate screens of the microbial metabolite library. Each data point represents a single compound and dashed lines indicate 20 % survival of each replicate, used as the threshold for activity (*i.e.* active ≤20 % survival, inactive >20 % survival). Compound classes are assigned a colour if at least 2 compounds from the structural class demonstrate activity based on the average survival. Single active compounds from a class have been grouped as ‘others’, whilst non-active compounds (NAs) appear in grey. **(b)** Compound classes with at least 2 active compounds against *T. gondii*, showing the proportion of active compounds in their classes and the total number of compounds in the respective classes within the library. Compound classes outlined in green showed the same activity in *P. falciparum*. **(c)** UpSet plot depicting the overlap between anti-toxoplasma activity of microbial metabolites with anti-tumour (NS-1 cell line) and cytotoxic (fibroblast cells) activity. The filled circles on the x-axis specify the overlapping activities and the height of the bars indicate the number of overlapping active compounds. The horizontal bars represent the total number of active compounds in each screen.

In equivalent 72 h screens against the *T. gondii* RH strain, we identified 149 compounds (18 %) that inhibited parasite growth by ≥ 80 %. Replicate testing had an R^2^ value of 0.7514 and the linear curve fitted between replicate data had a slope of 0.6148 (Fig. 2A). Two percent of compounds (16 compounds) produced inconsistent growth inhibition between replicates (Table S4). Compounds from these classes were not further examined against *T. gondii*, however one of these compounds belonging to the macrolide spinosyn subclass was further examined against *P. falciparum* where more than 50 % of the class was active against the parasite.

The 149 active compounds belonged to 57 chemical classes (Fig. 2A). Of these, 29 classes contained 2 or more active compounds, including 21 macrolides across 6 subclasses (Fig. 2B). There were 11 classes of which all the compounds in this library were active, including for several macrolide subclasses (bafilomycins, epothilones and venturicidins), the quinomycin peptides, aureolic acids, glutarimides, kibdelones, macrotetrolides, polyethers, trichothecenes, and tropones (Fig. 2B). Known inhibitors of *T. gondii* present in this library included the invasion inhibitor cytochalasin D and the polyether ionophore antibiotic monensin (Gubbels et al., 2003; Zhai et al., 2020).

Of the 149 anti-toxoplasma actives, 13 were selective for *T. gondii*, which included macrolide antibiotics in the leucomycin subclass (Fig. 2C). For example, leucomycin complex, also known as kitasamycin, is used as an antibacterial agent in animals and is related to spiramycin, which is clinically used to treat maternal toxoplasmosis during pregnancy. Of the compounds with activity against *T. gondii*, there were 136 compounds that had anti-tumour activity, including the kazusamycins belonging to the leptomycin class, which has previously been reported to inhibit tumour cells (Ando et al., 2006) (Fig. 2C). Additionally, 30 % of the anti-toxoplasma actives (45 compounds) also exhibited cytotoxic activity, including leptomycin A, an inhibitor of nuclear transport, demonstrating different exporter selectivity of the leptomycin class (Fig. 2C).

### 3.4 Evaluating the potency of actives against *T. gondii*

The compounds that had selective activity against *T. gondii* generally exhibited activity against *P. falciparum* too. We identified one compound (pladienolide B) active against *T. gondii* but not *P. falciparum* that had limited published information on its anti-toxoplasma activity.

Further analysis of the novel macrolide pladienolide B demonstrated that it was indeed highly potent against *T. gondii* in the low nanomolar range (IC_50_ = 6.7 ± 4.4 nM) (Table 2, Fig. S2). However, our cytotoxicity analysis against fibroblasts revealed it has an IC_50_ of 47 nM, resulting in a selectivity index of 7. This low selectivity index excluded it from being further characterised, despite its potency against *T. gondii*.

### 3.5 Comparison of antimalarial and anti-toxoplasma activity

Given their phylogenetic relationship and the fact that *P. falciparum* infects non-nucleated red blood cells whilst *T. gondii* infects any nucleated mammalian cell, comparing relative efficacy of specific compounds provides insight into shared mechanisms of action.

**Fig. 3:**
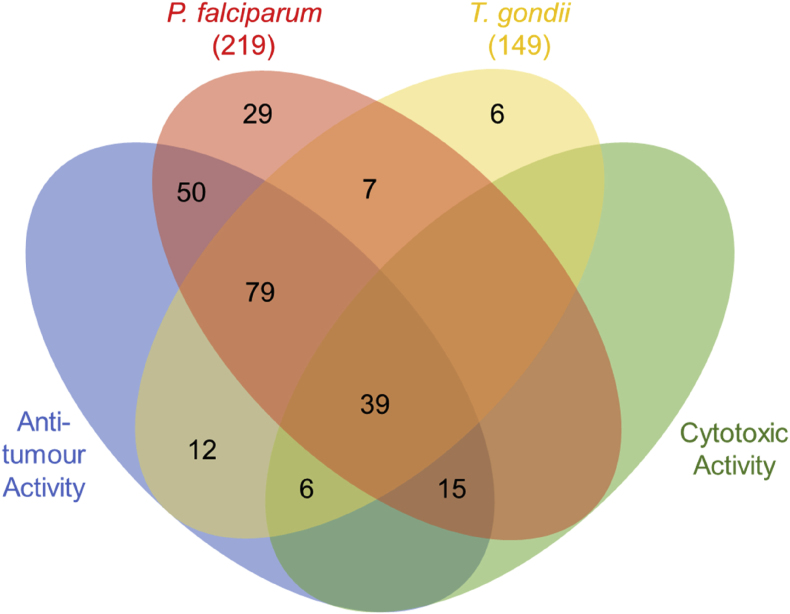
Active compounds against *P. falciparum* and *T. gondii*, and their anti-tumour and cytotoxic activity. Venn diagram depicting the overlap between antimalarial (*P. falciparum*) and anti-toxoplasma (*T. gondii*) activity of the active microbial metabolites with anti-tumour (NS-1 cell line) and cytotoxic (foreskin fibroblast cells) activity. The total number of active compounds against *P. falciparum* and *T. gondii* are indicated in parentheses.

There were 125 compounds that were active against both parasites (Fig. 3). Of the compound classes with at least two active compounds, there were nine compound classes where all examples in this library had activity against both parasites (Fig. 1, Fig. 2B). These included broadly active compounds (*e.g.* bafilomycins, macrotetrolides, quinomycins, trichothecenes) and compounds with established modes of action (*e.g.* aureolic acids, epothilones, venturicidins), with many of them having previously reported activity in *P. falciparum*, *T. gondii* or other parasitic protozoans (*e.g. Trypanosoma*, *Leishmania*) (Hauser et al., 2024; Taraschi et al., 1998). This included clinically used compounds, such as the polyether ionophores (*e.g.* monensin) used as veterinary drugs against coccidia (*e.g. Toxoplasma*, *Eimeria*, *Cryptosporidium*) (Lavine and Arrizabalaga, 2011; McDonald et al., 1990; McDougald, 1978). However, these classes also demonstrated anti-tumour activity; in fact, 118 of the 125 shared compounds in our screens exhibited anti-tumour activity (Fig. 3). Furthermore, 39 of these compounds also displayed cytotoxicity against fibroblasts (Fig. 3). This included the kibdelones, a family of heterocyclic polyketides that have received little attention (Ratnayake et al., 2007; Rujirawanich et al., 2016), with this being the first reported activity in *P. falciparum* and *T. gondii* (Fig. 1, Fig. 2B). Despite the cytotoxicity observed in our screen, these compounds have been reported to disrupt the actin cytoskeleton and may therefore be selective for cells that have a higher demand for actin remodelling, such as cancer cells and intracellular parasites (Gonzalez et al., 2009; Jiang et al., 2021; Rujirawanich et al., 2016).

Only 7 compounds were active selectively against *P. falciparum* and *T. gondii* (Fig. 3). These included 3 members of the cytochalasin family of actin polymerisation inhibitors; cytochalasin C, cytochalasin J and 19,20-epoxycytochalasin C. Whilst there appears to be no prior research on the activity of these cytochalasins against *P. falciparum* and *T. gondii*, the more common and potent members of the family (*e.g.* cytochalasins B and D) have been used extensively (Das et al., 2017; Dobrowolski and Sibley, 1996; Field et al., 1993; Oliveira-Lima et al., 2001). Furthermore, cytochalasins can affect biological processes unrelated to actin polymerisation (*e.g.* protein synthesis (Ornelles et al., 1986)). Therefore, based on these data, the increased antiparasitic selectivity of cytochalasin C, cytochalasin J and 19,20-epoxycytochalasin C could be a result of more specific binding to parasite actin for these analogues, but could also be due to a shift in the compounds’ target to one that is important for parasite growth. There were 3 macrolides that were exclusively active against both parasites. Lastly, the methylation inhibitor sinefungin, to which resistant *P. falciparum* and *T. gondii* parasites have been selected (Behnke et al., 2015; Inselburg, 1984), was also active.

Amongst the compounds with activity against both parasites and mammalian cells, several have no previously reported activity against these parasites and were found to have interesting potency and/or properties.

Cryptopleurine, a plant-derived phenanthroquinolizidine, displayed activity against both parasites and was the only representative of its compound class in this library. Dose-response curves demonstrated it had nanomolar potency against both apicomplexans (*P. falciparum* IC_50_ = 8 nM, *T. gondii* IC_50_ = 11 nM) (Table 2, Fig. S3), comparable to the *in vitro* activity of current clinically used antimalarial drugs. However, it also exhibited toxicity towards human fibroblasts (IC_50_ = 27 nM) resulting in a low selectivity index of 2.5, limiting prospects for further development in its current state.

A group of analogues that were further analysed against both parasites were the pleuromutilins. This class of drugs has antibacterial activity through inhibition of protein synthesis via binding to the 23S ribosomal RNA (Poulsen et al., 2001). Our screen consisted of seven analogues in this class, with activity observed for retapamulin, valnemulin, tiamulin and azamulin. Four of the analogues (retapamulin, valnemulin, pleuromutilin, tiamulin) were available for further characterisation. Retapamulin, used clinically as a topical treatment for human bacterial infections and predicted to target malaria parasites (Ramakrishnan et al., 2017), was active against both *P. falciparum* and *T. gondii* in our 72 h screens (Table 2). Our 120 h screens for *P. falciparum* indicated delayed-death activity for azamulin and tiamulin (Table 1), with valnemulin meeting two of our three criteria for delayed-death activity (Table S3). Valnemulin was also active against *T. gondii* in our 72 h screen (Table 2).

Two pleuromutilins, retapamulin and valnemulin, exhibited potent activity against *T. gondii* over 72 h with IC_50_ of 85 nM and 232 nM, respectively (Table 2, Fig. S4A and B). Furthermore, both analogues had selectivity indices greater than 100, indicating improved potency against *T. gondii* over the host cells (Fig. S4A). Conversely, none of the analogues were found to exhibit an IC_50_ of <1 μM against *P. falciparum* with 72 h treatments (Table 2, Fig. S4A and C). Since our 120 h screen detected activity of some analogues against *P. falciparum*, we undertook dose-response assays to assess the potential for delayed-death activity (Fig. S4A and C). The parent compound, pleuromutilin, did not display any activity, whilst tiamulin, which displayed delayed-death activity in our screen, appeared to have an IC_50_ close to 1 μM (Fig. S4C), indicating improvement in activity with longer treatment times was minimal. Valnemulin exhibited an IC_50_ of 64 nM and retapamulin an IC_50_ of 106 nM, suggesting that they exert a delayed-death like activity, as would be consistent with their reported mechanism of action in bacteria (Fig. S4A and C).

## 4 Discussion

With an urgent need for novel antimicrobial drugs to overcome emerging resistance and provide safe and efficacious treatments, exploring natural products that present a wide array of complex chemical scaffolds could help identify compounds with new mechanisms of action. Our screen of the BioAustralis Discovery Plates Series I library against the apicomplexan parasites *P. falciparum* and *T. gondii* has highlighted the potential of microbial metabolite compounds in the search for novel antiprotozoal drugs. Whilst this library contains many known compounds with established modes of action, a number of lesser-known compounds and novel analogues were identified as active against these protozoans. Though few of these may be promising for further development, they may offer the possibility to study parasite biology and gain insight into certain targets.

Although our understanding of the mechanisms of action of most microbial metabolites is partial at best, they represent the richest source of functionally active compounds available for discovery. Within a library of many known metabolites, high hit-rates are expected and the challenge lies in identifying the potent and selective leads. Our screen identified 27 and 18 % of compounds with fast-killing activity against *P. falciparum* and *T. gondii* parasites, respectively. The compound library was initially screened at 2 μg/mL which allowed for efficient screening across both parasite species whilst keeping DMSO concentrations low to avoid side effects and false positives. Given the variable molecular weights of natural products, this resulted in >90 % of compounds being screened at between 1 and 10 μM. Therefore, when assessing the IC_50_ of initial hits, it was not uncommon to subsequently determine that a compound had an IC_50_ greater than 1 μM, well above the activity range we were seeking to investigate further. This led to the early removal of the beauvericin, thielavin, padanamide and spinosyn classes from further structural binning and *in vitro* analysis.

Despite the lower than desired potency, some of these compounds may still hold potential. One such example is the family of beauvericin analogues. To our knowledge, this is the first report of their activity against *P. falciparum*, and of acyl-CoA:cholesterol acyltransferase inhibitors in *P. falciparum*. Although these beauvericin analogues were not as potent as clinically used antimalarial drugs, their activity is still an interesting finding. *Plasmodium* lacks a *de novo* cholesterol synthesis pathway and obtains cholesterol from the host, where it is stored as cholesteryl esters in lipid droplets (Hayakawa et al., 2020). These compounds may hold potential for combination therapies; statins, which affect cholesterol production, have been reported to have a synergistic effect with other antimalarial drugs and have displayed efficacy in rodent malaria models (Parquet et al., 2009, 2010; Souraud et al., 2012). These statins have exhibited a higher IC_50_ relative to the IC_50_ of some of the beauvericin analogues investigated here (Parquet et al., 2009, 2010). Furthermore, beauvericin has a reported activity around 1 μM in the parasitic protozoan *Leishmania*, which also takes up host cholesterol, and has demonstrated potency in *in vivo* models (Filho et al., 2024). Hence, these analogues may be worth further investigation in synergy assays with current antimalarials and those in development.

Amongst the compound classes that we further investigated, we identified compounds that were highly potent across four classes. Unfortunately, two of these individual compounds, pladienolide B and cryptopleurine, were also cytotoxic towards fibroblasts, making them unsuitable for further development into clinically used drugs. Pladienolide B, potent against *T. gondii*, was isolated in 2004 and is reported to exhibit anti-cancer properties by binding to the SF3b complex of the spliceosome thereby blocking it (Kotake et al., 2007; Mizui et al., 2004). Whilst this is the first report of pladienolide B's activity against *T. gondii*, there have been studies of other potent splicing inhibitors in *T. gondii* (Swale et al., 2022). Cryptopleurine, reported to inhibit eukaryotic protein translation and target the NF-κB pathway affecting cell survival (Bucher and Skogerson, 1976; Jin et al., 2012), was potent against *P. falciparum* and *T. gondii*. To our knowledge this is the first report of its activity against *P. falciparum* and *T. gondii*, however a recent study reported similar potencies against *P. falciparum* using an analogue extracted from a different plant species (Abdel-ٍٍ;Sattar et al., 2020). To be progressed further in their analysis as potential drugs, these compounds would need to be modified for significant improvement in their selectivity towards the parasites. Using an engineered *Streptomyces* lab strain, additional analogues of pladienolide B have been isolated with improved selectivity for a human tumour cell line over fibroblasts (Booth et al., 2020). Furthermore, pladienolide B has two analogues, E7107 and H3B-8800, which were progressed to phase I clinical trials for cancer, providing pharmacokinetic and toxicity data (Hong et al., 2013; Steensma et al., 2021). A study looking at the structure-activity relationship of cryptopleurine analogues against hepatitis C virus showed that this compound can be modified, with analogues showing up to a 5-fold increase in the selectivity index despite some loss in anti-viral activity (Wang et al., 2018). These studies provide data that can inform future research on modifications of these compounds for improved efficacy and safety.

In their current state, compounds that are not highly potent or exhibit cytotoxicity, can still be useful as tools for probing cellular mechanisms. A well-known example is staurosporine, a broad kinase inhibitor, which was also active in our screens. Whilst the broad inhibitory activity of staurosporine limits its clinical potential, it is used in research to understand signalling pathways and cellular processes (Belmokhtar et al., 2001; Rüegg and Burgess, 1989). Furthermore, it has also served as a lead compound to develop new kinase inhibitors (Wang et al., 2008). Similarly, the compounds in the current study that had limitations may be useful for studying their known targets. They may have the potential to be modified, as demonstrated by prior research, or their structure could inform us on the chemical moieties that confer potency thereby contributing to the development or modification of other compounds.

The thiazole peptide compounds we investigated proved they were potent and selective against *P. falciparum*. These compounds have predominantly been studied as antibacterial agents targeting protein synthesis. Our results showed that micrococcin P1 was highly potent against *P. falciparum*, as previously reported (Rogers et al., 1998). This is however the first report of the antimalarial activity of thiocillins I, II and III. Other thiazole peptides have been studied in *Plasmodium*. For example, thiostrepton exhibits an IC_50_ of approximately 10 μM, with its best analogues showing improved potency at 1 μM (Aminake et al., 2011; Schoof et al., 2010). Thiostrepton has been reported to exhibit potent activity against intraerythrocytic and transmission stage *Plasmodium* parasites through multiple modes of action, including targeting not only protein synthesis in the apicoplast but also the mitochondria, and interfering with the proteasome, thereby displaying fast-killing activity (Aminake et al., 2011; Azevedo et al., 2017; Bailly, 2022; Gupta et al., 2013; Schoof et al., 2010; Tarr et al., 2011). It is reasonable to assume that these thiocillins, which have not been extensively investigated, may function through similar mechanisms, though with 10-fold better potency than thiostrepton analogues. Thiostrepton, which also exhibits significant anti-cancer activity, is only used in veterinary applications in complex topical ointments to treat bacterial skin and eye infections (Bailly, 2022). Due to poor water solubility, formulation difficulties have restricted its development for human clinical use. This same property would present challenges in developing the thiocillin compounds examined here. Nevertheless, with almost half the drugs on the market and more than half the compounds in the discovery pipeline presenting poor water solubility, technologies have been developed for improved formulations (Xie et al., 2024) and efforts have been made to bioengineer more soluble derivatives. For example, the water-soluble semi-synthetic derivatives of nocathiacin, another thiazole peptide, have demonstrated potent activity against *P. falciparum* (Sharma et al., 2015). Therefore, given the significant research on thiostrepton against *Plasmodium*, and the potential to overcome pharmacokinetic obstacles, these thiocillin compounds are worth further investigation.

Interestingly, micrococcin P1, which was originally isolated over 70 years ago, was lost and research into this family of compounds was stalled until the discovery of the thiocillins almost 3 decades later (Shoji et al., 1976). This highlights the usefulness of the BioAustralis Discovery Plates compound library, containing rarer compounds that have had little opportunity to be investigated in the past.

A small selection of compounds were potential delayed-death inhibitors of *P. falciparum*, whereby they inhibited growth in the second round of replication instead of the first. These results aligned with existing knowledge and research on these compounds, with eight of the ten potential delayed-death inhibitors related to compounds with reported delayed-death activity. It is worth noting that the screening concentration and our 80 % growth inhibition threshold used to select for potent compounds, and thereby define criteria for delayed-death inhibitors, would have eliminated some potential delayed-death inhibitors. For example, we defined the criteria using azithromycin, clindamycin, doxycycline and spiramycin, which consistently exhibit a high IC_50_ concentration of at least 5 μM at 48–72 h and a low nanomolar range IC_50_ at more than 96 h (Wilson et al., 2013). However, some delayed-death inhibitors may be more potent in the first round of replication and therefore not meet our criteria. An example of such a compound in our screen was roxithromycin, which has a reported IC_50_ of approximately 4 μM at 48 h, down to 1–2 μM with increased treatment duration, resulting in more potency in the first cycle compared to other delayed-death macrolides, and thereby leading to its exclusion from our list of delayed-death inhibitors (Min et al., 2007). However, it is noticeable that half of the compounds which showed increased activity in the second cycle of growth, but were excluded from being grouped as delayed-death candidates based on our criteria, belonged to the same classes as known delayed-death inhibitors or have been reported to likely have this mechanism of action. Therefore, whilst our efforts to identify delayed-death inhibitors by grouping based on growth identified ten known or predicted apicoplast inhibitors, and a larger number of compounds with reported potential for delayed-death activity, this process did not result in identification of any potentially new apicoplast targeting drugs.

One of the compound classes that showed greater potency with longer treatment times in addition to fast-killing activity was the pleuromutilins, which in bacteria are known to inhibit protein synthesis (Poulsen et al., 2001). We followed up on several of these analogues and observed some interesting activity from two of the analogues, retapamulin and valnemulin, both semi-synthetic derivatives of pleuromutilin. We demonstrated that they exert potent fast-killing activity against *T. gondii* but had properties that resembled delayed-death activity against *P. falciparum*, suggesting that they may have different targets within these parasites. One possibility is that these compounds have targets in *T. gondii* that lead to rapid death in addition to the inhibition of apicoplast processes that result in a delayed-death phenotype. A recent study reported apicoplast loss in *T. gondii* induced by retapamulin, however they also observed activity at 48 h, supporting the idea of multiple targets (Dos Santos et al., 2023). Though retapamulin has been predicted to target malaria parasites (Ramakrishnan et al., 2017), this is the first report demonstrating the activity of these analogues in *P. falciparum* and in comparison with *T. gondii*. Retapamulin was the first pleuromutilin approved for human use as a topical antibiotic (Jones et al., 2006). However, it is not suitable for systemic use due to extensive metabolism and poor bioavailability (Sun et al., 2018). There is however another pleuromutilin, lefamulin, that is used systemically in humans (Eyal et al., 2016), raising the question of whether the pharmacokinetic properties of retapamulin can be improved. Meanwhile, valnemulin is available for veterinary use to control gastrointestinal and respiratory infections in animals (Stingelin et al., 2022). Furthermore, both analogues, retapamulin and valnemulin, have no clinically relevant cross-resistance to existing drugs due to a unique binding site on the ribosome (Yan et al., 2006). The differing activity we observed between *P. falciparum* and *T. gondii*, and their clinical application, makes this family of compounds very intriguing for further investigation against these parasites, and may offer the opportunity to repurpose and improve known compounds.

Drug repurposing is an alternative and efficient pathway to developing new treatments. The possibility to repurpose compounds may also apply to compounds in this library that we did not investigate further. Many of the compounds are well-characterised, especially in bacteria, though they may not have had their antimalarial and anti-toxoplasma activity explored in depth and may thus act as a starting point for the development of new antiprotozoal compounds. Additionally, they may have analogues that have not been extensively studied, such as the micrococcin P1 thiocillin analogues. Therefore, our screen of this library can provide summary data on the activity of many known compounds against *P. falciparum* and *T. gondii* parasites worth further investigation.

Several studies have highlighted the potential of natural products, using extracts or pure compounds, as antimalarial and anti-toxoplasma compounds (Jiang et al., 2024; Pérez-Moreno et al., 2016). The BioAustralis Discovery Plates Series I collection comprises microbial metabolites sourced mainly from Australia. This is the first time it has been screened against *P. falciparum* and *T. gondii*, confirming the activity of known active compounds but also identifying novel compounds with potential. The screening of natural products provides a valuable tool in the search for new therapeutics, as the diverse chemistry and biology increase the likelihood of identifying physiologically active compounds, which often offer evolutionary advantages to the producing organisms. Exploring novel chemical scaffolds could help identify compounds with new modes of action. This is in contrast to chemical libraries, which are based on the iterative synthesis of known scaffolds. However, using natural product libraries comes with challenges. These include difficulties in culturing microorganisms and obtaining sufficient yields, as well as the required expertise in determining and isolating pure compounds. Natural product compounds can also face challenges due to their size or chemistry. Furthermore, modification and optimisation strategies such as individualised manipulation or structure-activity relationship analysis can be challenging. Nevertheless, many natural products have gone on to be modified successfully for properties that are of benefit for clinical use, such as the antimalarial artemisinin (Lin et al., 1987) and the antibiotic erythromycin (Jelić and Antolović, 2016) to name a few.

## 5 Conclusion

Our screens of the BioAustralis Discovery Plates Series I compound library demonstrate the potential of developing antimalarial and anti-toxoplasma drugs from microbial metabolites. We identified a high number of active compounds, highlighting the activity of many compound classes. From a small selection of compounds, we identified compounds with IC_50_ values in the low nanomolar range (*e.g.* thiocillins, pleuromutilins, pladienolide B, cryptopleurine) that can be prioritised for further investigation. In addition to exploring their mechanisms of action in the parasites and examining the *in vivo* potency, structure-activity relationship analysis is necessary to inform their optimisation for improved specificity and bioavailability. We also identified compounds in the high nanomolar range, with the potential of many of the other actives falling in the low micromolar range. These actives may still prove to be interesting and could serve as a starting point for development of other compounds but may also be worth exploring in combination therapies with existing drugs. Additionally, the combined data screens provide comparison data on possible shared targets between these parasites, as well as prioritisation of compounds based on known activity, especially since more compound data is available for *P. falciparum* than *T. gondii*. In summary, the compounds identified in the present screen provide valuable information for antiprotozoal drug discovery from microbial sources.

## CRediT authorship contribution statement

**Maria R. Gancheva:** Writing – review & editing, Writing – original draft, Visualization, Methodology, Investigation, Formal analysis, Data curation, Conceptualization. **Emma Y. Mao:** Writing – review & editing, Investigation, Formal analysis. **Ornella Romeo:** Writing – review & editing, Methodology. **Daniel Vuong:** Writing – review & editing, Data curation. **Ryan O'Handley:** Writing – review & editing, Resources. **Stephen W. Page:** Writing – review & editing, Conceptualization. **Ernest Lacey:** Writing – review & editing, Resources, Data curation, Conceptualization. **Danny W. Wilson:** Writing – review & editing, Supervision, Resources, Funding acquisition, Conceptualization.

## Note

Supplementary data associated with this article.

## Funding source

This work was conducted as part of the Australian Research Council Industrial Transformation Training Centre for Environmental and Agricultural Solutions to Antimicrobial Resistance (IC220100050) and funded by its Partners and the Australian Government. Support was also received through a Hospital Research Foundation Collaborative Research Grant (2021/49-QA25312), Australia. EYM was supported by The Muriel Faulkner Simms Research Scholarship in Therapeutics, DWW by a Hospital Research Foundation Fellowship (2019/41–83100), EL and SWP by an Australian Research Council Cooperative Research Centre Project (CRC-P) Grant.

## Conflict of interest statement

Authors MRG, EYM, OR, DV, RO, SWP, EL, and DWW declare no financial or non-financial competing interests. Author SWP holds shares in Advanced Veterinary Therapeutics, Luoda Pharma and Neoculi, and has previously acted as a paid consultant for Zoetis, Boehringer Ingleheim, Elanco, Virbac and Ceva but declares no financial or non-financial competing interests.
