# Supplementary material for: Screening and hit evaluation of a microbial metabolite library against the pathogenic Plasmodium falciparum and Toxoplasma gondii parasites
Source: Int J Parasitol Drugs Drug Resist. 2025 Aug 5;29:100606. doi: 10.1016/j.ijpddr.2025.100606 (PMC12396409; doi:10.1016/j.ijpddr.2025.100606)
Supplement: Multimedia component 2 [file mmc2.docx]

*SUPPLEMENTARY INFORMATION*

**Screening and hit evaluation of a microbial metabolite library against the pathogenic *Plasmodium falciparum* and *Toxoplasma gondii* parasites**

Maria R. Gancheva^a,b,c,*^, Emma Y. Mao^a,b^, Ornella Romeo^a^, Daniel Vuong^d^, Ryan O’Handley^c^, Stephen W. Page^e^, Ernest Lacey^d^, Danny W. Wilson^a,b,f,*^

^a^Research Centre for Infectious Diseases, School of Biological Sciences, The University of Adelaide, Adelaide 5005, South Australia, Australia.

^b^ARC Training Centre for Environmental and Agricultural Solutions to Antimicrobial Resistance (CEAStAR), St Lucia 4072, Queensland, Australia.

^c^School of Animal and Veterinary Sciences, The University of Adelaide, Roseworthy 5371, South Australia, Australia.

^d^Microbial Screening Technologies Pty Ltd, Smithfield 2164, New South Wales, Australia.

^e^Neoculi Pty Ltd, Newtown 2042, New South Wales, Australia.

^f^Burnet Institute, Melbourne 3004, Victoria, Australia.

*Corresponding Author:

E-mail: [danny.wilson@adelaide.edu.au](mailto:danny.wilson@adelaide.edu.au), [maria.gancheva@adelaide.edu.au](mailto:maria.gancheva@adelaide.edu.au)

**Table S2.** Compounds with inconsistent growth inhibition between replicates against *P. falciparum* at 72 h.

| **Compound** | **Class** | **Replicate**  **1** | **Replicate**  **2** | **Mean** | **Number of Additional Active Compounds in Class** |
| --- | --- | --- | --- | --- | --- |
| Bischloroanthra-benzoxocinone | Anthrabenzoxo-cinone | 16.72 | 45.58 | 31.15 | 0 |
| Benzomalvin B | Benzodiazepine | 1.98 | 79.80 | 40.89 | 0 |
| Eupenifeldin | Bistropolone | 0.51 | 57.11 | 28.81 | 1 |
| Sinapic acid | Cinnamic acid | 90.37 | 1.58 | 45.98 | 0 |
| Chrysomycin A | Gilvocarcin | 16.21 | 57.66 | 36.94 | 1 |
| Nybomycin | Heteroaromatic | 45.31 | 18.57 | 31.94 | 0 |
| Tilmicosin phosphate | Leucomycin | 4.49 | 39.64 | 22.07 | 0 |
| Lydicamycin | Lydicamycin | 68.30 | 5.09 | 36.70 | 0 |
| Conglobatin | Macrodiolide | 86.29 | 18.30 | 52.30 | 1 |
| Milbemycin A4 oxime | Milbemycin | 26.87 | 18.08 | 22.48 | 12 |
| Nemadectin | Milbemycin | 30.14 | 13.82 | 21.98 | 12 |
| Milbemycin A3 | Milbemycin | 47.24 | 1.70 | 24.47 | 12 |
| Ivermectin B1a monosaccharide | Milbemycin | 84.82 | 14.34 | 49.58 | 12 |
| Oligomycin D | Oligomycin | 16.24 | 27.09 | 21.67 | 4 |
| Chevalone B | Pyrone | 12.18 | 38.64 | 25.41 | 1 |
| Territrem A | Pyrone | 14.32 | 84.62 | 49.47 | 1 |
| Methylcanidine | Quinolizidine | 14.79 | 27.45 | 21.12 | 1 |
| Umirolimus | Tacrolimus | 19.04 | 26.75 | 22.90 | 1 |
| Ikarugamycin | Tetramic acid | 84.47 | 12.14 | 48.31 | 0 |

Compounds in; green belong to classes that contain >1 other active compound, yellow belong to classes that contain one other active compound, and red belong to classes that do not have any active compounds.

**Table S3.** Potential delayed-death candidates against *P. falciparum*.

| **Compound** | **Class** | **1) Growth (%)**  **at 72 h** | **2) Growth (%)**  **at 120 h** | **3) Fold change**  **(72 h/120 h)** |
| --- | --- | --- | --- | --- |
| Florfenicol | Amphenicol | 62.44 | 10.76 | 5.8 |
| Thiamphenicol | Amphenicol | 49.53 | 19.75 | 2.5 |
| Novobiocin | Coumarin | 81.42 | 14.18 | 5.7 |
| Virginiamycin complex | Depsipeptide | 47.81 | 18.06 | 2.6 |
| Azithromycin | Erythromycin | 56.17 | 8.77 | 6.4 |
| Davercin | Erythromycin | 62.94 | 13.05 | 4.8 |
| Gamithromycin | Erythromycin | 58.53 | 15.46 | 3.8 |
| Roxithromycin | Erythromycin | 30.91 | 8.49 | 3.6 |
| Tulathromycin | Erythromycin | 33.53 | 16.75 | 2.0 |
| Chrysomycin A | Gilvocarcin | 36.94 | -1.07 | -34.7 |
| Nybomycin | Heteroaromatic | 31.94 | 16.54 | 1.9 |
| Indolmycin | Indole | 44.88 | 17.39 | 2.6 |
| Isoreserpiline | Indole alkaloid | 24.60 | 4.30 | 5.7 |
| Josamycin (Leucomycin A3) | Leucomycin | 53.50 | 10.08 | 5.3 |
| Leucomycin A1 | Leucomycin | 58.63 | 11.56 | 5.1 |
| Leucomycin A13 | Leucomycin | 33.20 | 9.02 | 3.7 |
| Leucomycin A4 | Leucomycin | 46.88 | 1.51 | 31.0 |
| Leucomycin A5 | Leucomycin | 66.05 | 12.18 | 5.4 |
| Spiramycin I | Leucomycin | 71.72 | 11.98 | 6.0 |
| Tilmicosin | Leucomycin | 43.10 | 14.50 | 3.0 |
| Tilmicosin phosphate | Leucomycin | 22.07 | 7.83 | 2.8 |
| Tylosin | Leucomycin | 39.25 | 17.27 | 2.3 |
| Clindamycin 2-phosphate | Lincomycin | 67.91 | 9.80 | 6.9 |
| Clindamycin hydrochloride | Lincomycin | 39.99 | 2.75 | 14.5 |
| Lincomycin | Lincomycin | 54.39 | 14.14 | 3.8 |
| Alamethicin F50 | Linear peptide | 22.03 | 3.45 | 6.4 |
| 4-O-methylammocidin | Macrocyclic lactone | 43.16 | 13.10 | 3.3 |
| Eprinomectin B1a | Milbemycin | 42.60 | 19.45 | 2.2 |
| Milbemycin A3 | Milbemycin | 24.47 | 17.63 | 1.4 |
| Milbemycin A4 oxime | Milbemycin | 22.48 | 12.31 | 1.8 |
| Milbemycin oxime | Milbemycin | 23.75 | 3.08 | 7.7 |
| Nemadectin | Milbemycin | 21.98 | 11.78 | 1.9 |
| Oligomycin D | Oligomycin | 21.67 | 14.87 | 1.5 |
| Phloroglucinol | Phenol | 51.22 | 13.22 | 3.9 |
| Citreoindole | Piperazine | 27.30 | 9.52 | 2.9 |
| Azamulin | Pleuromutilin | 61.19 | 8.89 | 6.9 |
| Tiamulin | Pleuromutilin | 55.02 | 7.81 | 7.0 |
| Valnemulin | Pleuromutilin | 50.06 | 13.41 | 3.7 |
| Methylcanidine | Quinolizidine | 21.12 | 11.61 | 1.8 |
| Rebeccamycin | Staurosporine | 21.58 | 18.40 | 1.2 |
| Chevalone B | Terpenoid (mero) | 25.41 | 17.52 | 1.5 |
| Helvolic acid | Terpenoid (tri) | 56.73 | 8.14 | 7.0 |
| Doxycycline | Tetracycline | 48.60 | 7.68 | 6.3 |
| Epitetracycline hydrochloride | Tetracycline | 57.67 | 14.73 | 3.9 |
| Oxytetracycline | Tetracycline | 47.36 | 15.15 | 3.1 |
| Actinotetraose Hexatiglate | Tetrasaccharide | 46.54 | 19.22 | 2.4 |
| Eupenifeldin | Tropone | 28.81 | 14.46 | 2.0 |
| Dalfopristin mesylate | Streptogramin | 45.58 | 5.60 | 8.1 |
| Quinupristin-Dalfopristin mesylate complex | Streptogramin | 33.15 | 7.64 | 4.3 |

Delayed-death compounds were defined as those that: 1) exhibited ≥40 percent growth at 72 h, but 2) ≤20 percent growth at 120 h, with 3) a difference of at least 5-fold increase in potency from 1 to 2 cycles of intraerythrocytic replication (from 72 to 120 h). Compounds highlighted in yellow are known delayed-death inhibitors used to define the criteria for delayed-death. Compounds highlighted in green are the delayed-death candidates.


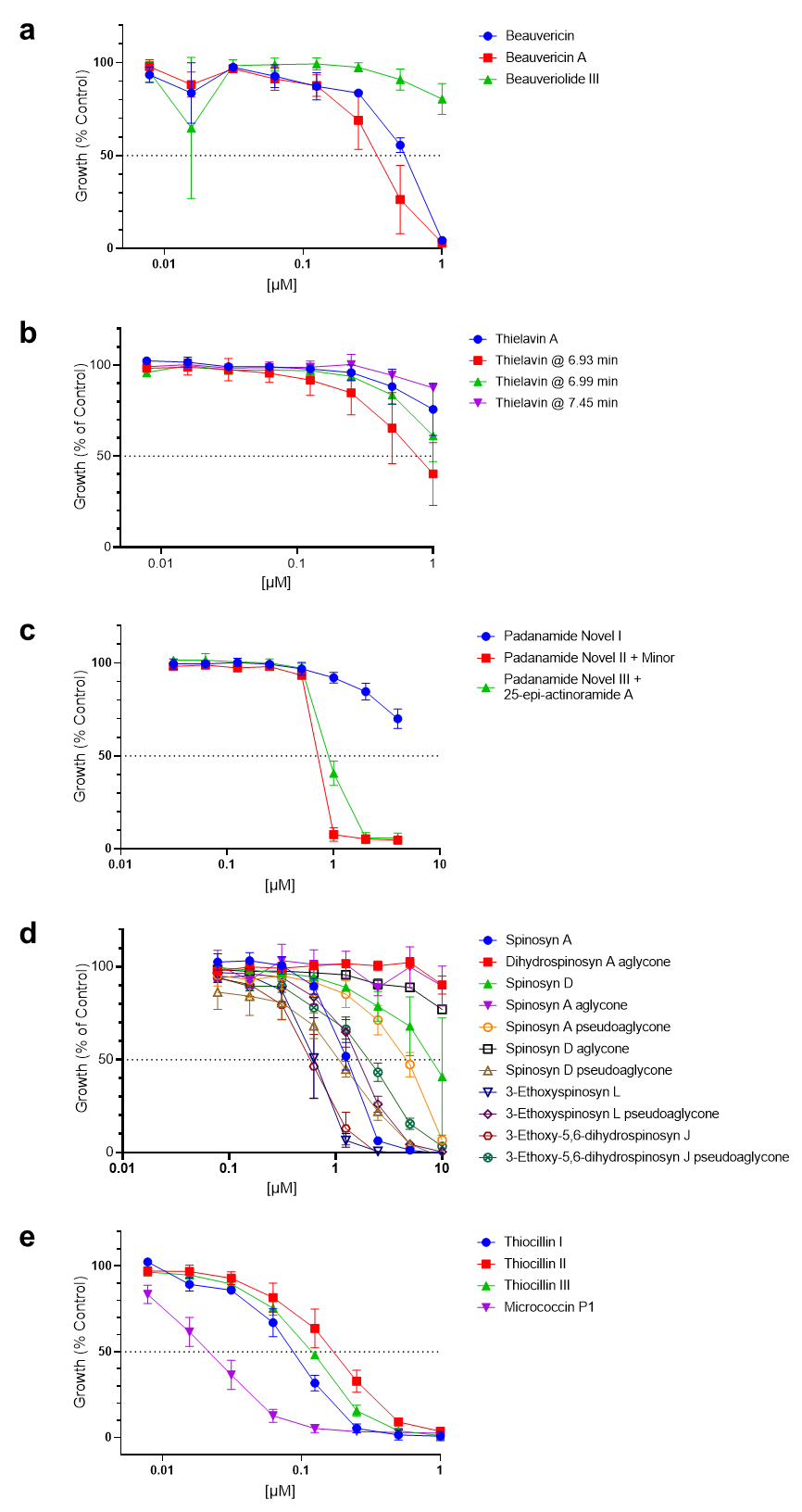


**Figure S1.** Dose-response curves used to calculate the IC_50_ of selected compounds against *P. falciparum*. Data represents mean ± S.D., n ≥ 3.

**Table S4.** Compounds with inconsistent growth inhibition between replicates against *T. gondii* at 72 h.

| **Compound** | **Class** | **Replicate 1** | **Replicate 2** | **Mean** | **Number of Additional Active Compounds in Class** |
| --- | --- | --- | --- | --- | --- |
| Novobiocin | Aminocoumarin | 9.17 | 93.65 | 51.41 | 0 |
| Antimycin A3 | Antimycin | 30.27 | 12.84 | 21.56 | 3 |
| Penicolinate A | Bis-picolinic ester | 16.04 | 73.01 | 44.53 | 0 |
| Enniatin complex | Depsipeptide | 11.58 | 79.82 | 45.70 | 6 |
| Chrysomycin B | Gilvocarcin | 25.01 | 17.10 | 21.06 | 1 |
| Hodgkinsine B | Indole Alkaloid | 8.86 | 59.70 | 34.28 | 0 |
| Josamycin (Leucomycin A3) | Leucomycin | 18.33 | 23.52 | 20.93 | 2 |
| Leucomycin A4 | Leucomycin | 19.43 | 27.43 | 23.43 | 2 |
| Ophiobolin C | Ophiobolin | 7.63 | 89.80 | 48.72 | 2 |
| Prodigiosin | Pyrrole-derivative | 39.74 | 11.46 | 25.60 | 5 |
| Psychotridine | Pyrrole-derivative | 17.15 | 61.99 | 39.57 | 5 |
| Roseoflavin | Riboflavin | 40.04 | 16.23 | 28.14 | 0 |
| Spinosyn A 17-pseudoaglycone | Spinosyn | 50.49 | 9.69 | 30.09 | 0 |
| Oxostaurosporine, 7- | Staurosporine | 57.26 | 18.54 | 37.90 | 5 |
| Minocycline HCl | Tetracycline | 5.81 | 37.51 | 21.66 | 0 |
| Deoxyviolacein | Violacein | 14.71 | 26.55 | 20.63 | 0 |

Compounds in; green belong to classes that contain >1 other active compound, yellow belong to classes that contain one other active compound, and red belong to classes that do not have any active compounds.

**Figure S2.** Dose-response curve used to calculate the IC_50_ of pladienolide B against *T. gondii*. Data represents mean ± S.D., n ≥ 3.

**Figure S3.** Dose-response curves used to calculate the IC_50_ of cryptopleurine against *P. falciparum* and *T. gondii*. Data represents mean ± S.D., n ≥ 3.


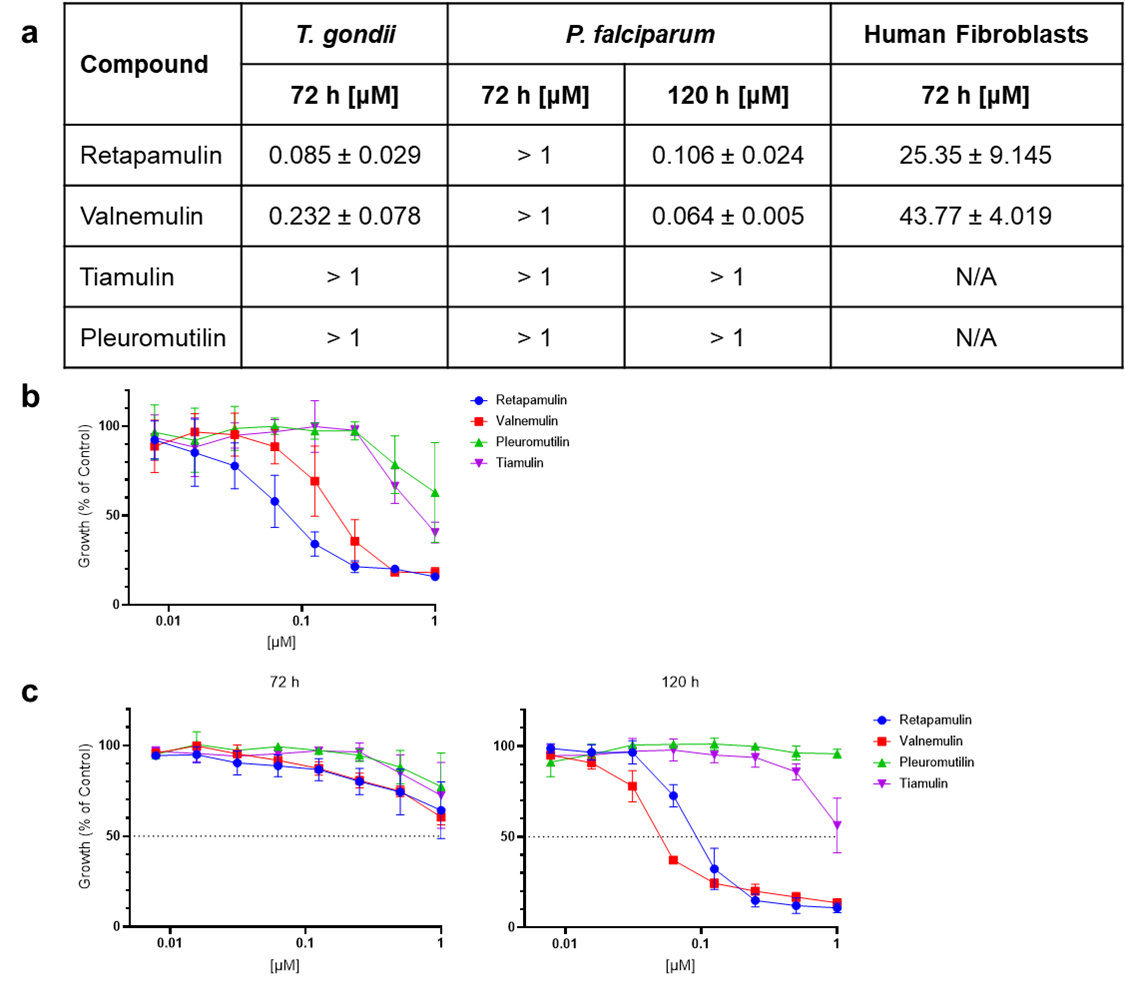


**Figure S4.** Dose-response curves and IC_50_ of the pleuromutilin analogues against *P. falciparum* and *T. gondii*. **(a)** IC_50_ values for *T. gondii* at 72 h, *P. falciparum* at 72 and 120 h, and human fibroblasts at 72 h. **(b)** Dose-response curve for *T. gondii*. **(c)** Dose-response curves for *P. falciparum*. Data represents mean ± S.D., n ≥ 3.
